# (*E*)-1-(3-Eth­oxy-2-hy­droxy­benzyl­idene)thio­semicarbazide

**DOI:** 10.1107/S1600536812000487

**Published:** 2012-01-11

**Authors:** Amir Adabi Ardakani, Hadi Kargar, Reza Kia, Muhammad Nawaz Tahir

**Affiliations:** a Ardakan Branch, Islamic Azad University, Ardakan, Iran; b Department of Chemistry, Payame Noor University, PO BOX 19395-3697 Tehran, I. R. of IRAN; c X-ray Crystallography Lab., Plasma Physics Research Center, Science and Research Branch, Islamic Azad University, Tehran, Iran; d Department of Chemistry, Science and Research Branch, Islamic Azad University, Tehran, Iran; e Department of Physics, University of Sargodha, Punjab, Pakistan

## Abstract

The title compound, C_10_H_13_N_3_O_2_S, crystallizes with two independent mol­ecules (*A* and *B*) in the asymmetric unit. In the crystal, the *A* and *B* mol­ecules are linked *via* pairs of N—H⋯O and O—H⋯S hydrogen bonds, forming dimers with *R*_2_^2^(14) and *R*_2_^2^(6) ring motifs. These dimers are linked *via* a pair of N—H⋯S hydrogen bonds with an *R*_2_^2^(8) ring motif, forming chains propagating along the *c*-axis direction. The crystal was refined as an inversion twin with a final BASF ratio of 0.54 (11):0.46 (11).

## Related literature

For standard bond lengths, see: Allen *et al.* (1987 ▶). For hydrogen-bond motifs, see: Bernstein *et al.* (1995 ▶). For background to thio­semicarbazones in coordination chemistry, see: Casas *et al.* (2000 ▶). For their biological applications, see: for example, Maccioni *et al.* (2003 ▶); Ferrari *et al.* (2000 ▶). For related structures, see: Kargar *et al.* (2010**a* ▶,b* ▶).
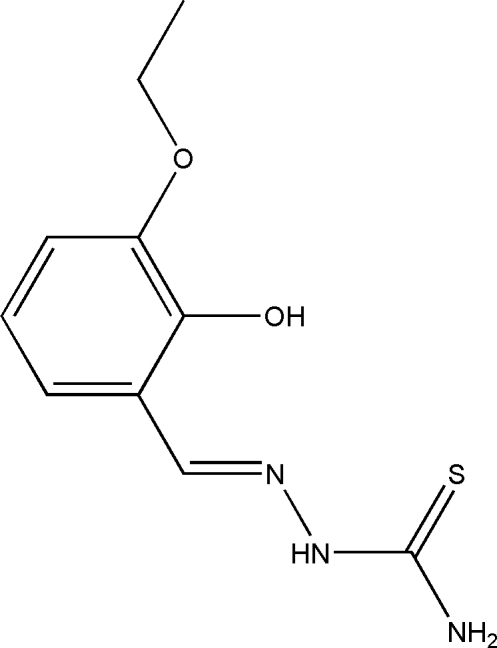


## Experimental

### 

#### Crystal data

C_10_H_13_N_3_O_2_S
*M* *_r_* = 239.29
Monoclinic, 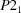

*a* = 6.0728 (3) Å
*b* = 16.1595 (8) Å
*c* = 12.8490 (6) Å
β = 90.238 (3)°
*V* = 1260.91 (11) Å^3^
*Z* = 4
Mo *K*α radiation
μ = 0.25 mm^−1^
*T* = 291 K
0.24 × 0.14 × 0.08 mm

#### Data collection

Bruker SMART APEX CCD area-detector diffractometer
Absorption correction: multi-scan (*SADABS*; Bruker, 2005 ▶) *T*_min_ = 0.800, *T*_max_ = 0.926
12062 measured reflections
5428 independent reflections
2303 reflections with *I* > 2σ(*I*)
*R*_int_ = 0.075

#### Refinement

*R*[*F* ^2^ > 2σ(*F* ^2^)] = 0.056
*wR*(*F* ^2^) = 0.119
*S* = 0.92
5428 reflections
293 parameters
1 restraint
H-atom parameters constrained
Δρ_max_ = 0.21 e Å^−3^
Δρ_min_ = −0.21 e Å^−3^
Absolute structure: Flack (1983 ▶), 2232 Friedel pairs
Flack parameter: 0.54 (11)

### 

Data collection: *APEX2* (Bruker, 2005 ▶); cell refinement: *SAINT* (Bruker, 2005 ▶); data reduction: *SAINT*; program(s) used to solve structure: *SHELXS97* (Sheldrick, 2008 ▶); program(s) used to refine structure: *SHELXL97* (Sheldrick, 2008 ▶); molecular graphics: *SHELXTL* (Sheldrick, 2008 ▶); software used to prepare material for publication: *SHELXTL* and *PLATON* (Spek, 2009 ▶).

## Supplementary Material

Crystal structure: contains datablock(s) global, I. DOI: 10.1107/S1600536812000487/su2360sup1.cif

Structure factors: contains datablock(s) I. DOI: 10.1107/S1600536812000487/su2360Isup2.hkl

Supplementary material file. DOI: 10.1107/S1600536812000487/su2360Isup3.cml

Additional supplementary materials:  crystallographic information; 3D view; checkCIF report

## Figures and Tables

**Table 1:** Hydrogen-bond geometry (Å, °)

*D*—H⋯*A*	*D*—H	H⋯*A*	*D*⋯*A*	*D*—H⋯*A*
O1—H1*O*⋯S2^i^	0.83	2.53	3.180 (4)	135
O3—H3*O*⋯S1^ii^	0.83	2.43	3.143 (4)	145
N2—H2*N*⋯O3^i^	0.90	2.20	2.954 (6)	142
N5—H5*N*⋯O1^ii^	0.87	2.17	3.009 (5)	160
N3—H3*NB*⋯S2^iii^	0.90	2.53	3.403 (4)	166
N6—H6*NB*⋯S1^iv^	0.88	2.55	3.398 (5)	161

Symmetry codes: (i) 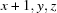; (ii) 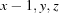; (iii) 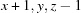; (iv) 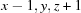.

**Fig. 1.**
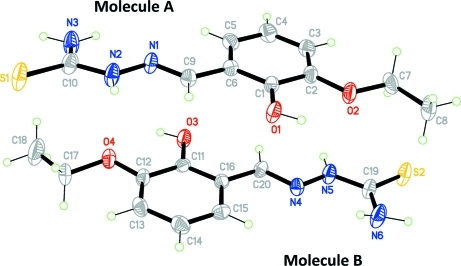
The molecular structure of the two independent molecules of the title compound, showing 40% probability displacement ellipsoids and the atomic numbering.

**Fig. 2.**
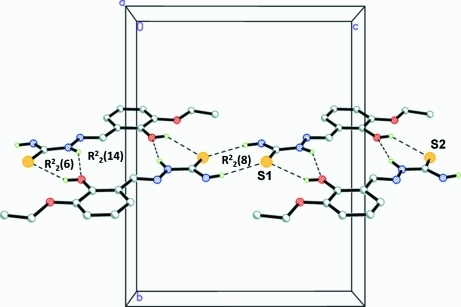
A partial crystal packing diagram of the title compound, viewed down the a-axis, showing a one-dimensional extended chain along the c-axis formed via intermolecular O—H···S, N—H···O, and N—H···S hydrogen bonds [dashed lines; see Table 1 for details; only the H atoms involved in these interactions are shown].

## supplementary crystallographic information

### Comment

Thiosemicarbazones constitute an important class of N,S donor ligands due to their propensity to react with a wide range of metals (Casas *et al.*, 2000). Thiosemicarbazones exhibit various biological activities and have therefore attracted considerable pharmaceutical interest (Maccioni *et al.*, 2003; Ferrari *et al.*, 2000). Herein, we report on the crystal structure of the new title thiosemicarbazone compound.

The title compound crystallized with two independent molecules (A and B) in the asymmetric unit, Fig. 1. The bond lengths (Allen *et al.*, 1987) and angles are within the normal ranges and are comparable to those observed for related structures (Kargar *et al.*, 2010**a*,b*).

In the crystal, the A and B molecules are linked *via* pairs of N-H···O and O-H···S hydrogen bonds (Table 1 and Fig. 2) to form dimers, with R^2^_2_(14) and R^2^_2_(6) ring motifs (Bernstein *et al.*, 1995). These dimers are further linked *via* a pair of N-H···S hydrogen bonds, with an R^2^_2_(8) ring motif, to form chains that extend in direction [0 0 1] (Table 1 and Fig. 2).

The crystal was refined as an inversion twin with a final refined BASF ratio of 0.54 (11)/0.46 (11) for 2232 Friedel pairs.

### Experimental

A mixture of 3-ethoxysalicylalehyde (0.01 mol) and hydrazinecarbothioamide (0.01 mol) in 20 ml of ethanol was refluxed for about 2 h. On cooling, the solid separated was filtered and recrystallized from ethanol. Colourless plate-like crystals of the title compound, suitable for X-ray diffraction, were obtained by slow evaporation of a solution in ethanol.

### Refinement

O- and N-bound H atoms were located in a difference Fourier map and were initially refined with the O-H and N-H distances restrained to 0.82 (2) and 0.86 (2) Å, respectively. In the final cycles of refinement they were constrained to ride on their parent atoms with U_iso_(H) = 1.5U_eq_(O) and 1.2U_eq_(N), respectively. The C-bound H-atoms were included in calculated positions and treated as riding atoms: C—H = 0.93, 0.96 and 0.97 Å for CH, CH_3_ and CH_2_ H-atoms, respectively, with U_iso_ (H) = k x U_eq_(C), where k = 1.5 for CH_3_ H-atoms, and k = 1.2 for all other H-atoms. The crystal was refined as an inversion twin with a final refined BASF ratio of 0.54 (11)/0.46 (11) for 2232 Friedel pairs.

### Crystal data

**Table:**

C_10_H_13_N_3_O_2_S	*F*(000) = 504
*M**_r_* = 239.29	*D*_x_ = 1.261 Mg m^−^^3^
Monoclinic, *P*2_1_	Mo *K*α radiation, λ = 0.71073 Å
Hall symbol: P 2yb	Cell parameters from 2525 reflections
*a* = 6.0728 (3) Å	θ = 2.5–29.5°
*b* = 16.1595 (8) Å	µ = 0.25 mm^−^^1^
*c* = 12.8490 (6) Å	*T* = 291 K
β = 90.238 (3)°	Plate, colourless
*V* = 1260.91 (11) Å^3^	0.24 × 0.14 × 0.08 mm
*Z* = 4	

### Data collection

**Table:**

Bruker SMART APEX CCD area-detector diffractometer	5428 independent reflections
Radiation source: fine-focus sealed tube	2303 reflections with *I* > 2σ(*I*)
graphite	*R*_int_ = 0.075
φ and ω scans	θ_max_ = 28.3°, θ_min_ = 1.6°
Absorption correction: multi-scan (*SADABS*; Bruker, 2005)	*h* = −8→7
*T*_min_ = 0.800, *T*_max_ = 0.926	*k* = −21→19
12062 measured reflections	*l* = −17→17

### Refinement

**Table:**

Refinement on *F*^2^	Hydrogen site location: inferred from neighbouring sites
Least-squares matrix: full	H-atom parameters constrained
*R*[*F*^2^ > 2σ(*F*^2^)] = 0.056	*w* = 1/[σ^2^(*F*_o_^2^) + (0.0341*P*)^2^] where *P* = (*F*_o_^2^ + 2*F*_c_^2^)/3
*wR*(*F*^2^) = 0.119	(Δ/σ)_max_ < 0.001
*S* = 0.92	Δρ_max_ = 0.21 e Å^−^^3^
5428 reflections	Δρ_min_ = −0.21 e Å^−^^3^
293 parameters	Extinction correction: *SHELXL97* (Sheldrick, 2008), Fc^*^=kFc[1+0.001xFc^2^λ^3^/sin(2θ)]^-1/4^
1 restraint	Extinction coefficient: 0.0087 (9)
Primary atom site location: structure-invariant direct methods	Absolute structure: Flack (1983), 2232 Friedel pairs
Secondary atom site location: difference Fourier map	Flack parameter: 0.54 (11)

### Special details

**Table:**

Geometry. All esds (except the esd in the dihedral angle between two l.s. planes) are estimated using the full covariance matrix. The cell esds are taken into account individually in the estimation of esds in distances, angles and torsion angles; correlations between esds in cell parameters are only used when they are defined by crystal symmetry. An approximate (isotropic) treatment of cell esds is used for estimating esds involving l.s. planes.
Refinement. Refinement of F^2^ against ALL reflections. The weighted R-factor wR and goodness of fit S are based on F^2^, conventional R-factors R are based on F, with F set to zero for negative F^2^. The threshold expression of F^2^ > 2sigma(F^2^) is used only for calculating R-factors(gt) etc. and is not relevant to the choice of reflections for refinement. R-factors based on F^2^ are statistically about twice as large as those based on F, and R- factors based on ALL data will be even larger.

### Fractional atomic coordinates and isotropic or equivalent isotropic displacement parameters (Å^2^)

**Table:**

	*x*	*y*	*z*	*U*_iso_*/*U*_eq_	
C1	0.2843 (8)	0.3987 (3)	1.0328 (4)	0.0372 (13)	
C2	0.1042 (9)	0.3625 (4)	1.0844 (4)	0.0424 (15)	
C3	−0.0613 (8)	0.3257 (4)	1.0275 (4)	0.0485 (16)	
H3	−0.1804	0.3020	1.0617	0.058*	
C4	−0.0523 (9)	0.3236 (4)	0.9203 (4)	0.0562 (16)	
H4	−0.1637	0.2980	0.8824	0.067*	
C5	0.1241 (9)	0.3600 (3)	0.8695 (4)	0.0478 (15)	
H5	0.1287	0.3594	0.7972	0.057*	
C6	0.2951 (8)	0.3976 (3)	0.9255 (4)	0.0381 (13)	
C7	−0.0470 (9)	0.3298 (4)	1.2529 (4)	0.062 (2)	
H7A	−0.0526	0.2708	1.2390	0.074*	
H7B	−0.1900	0.3534	1.2367	0.074*	
C8	0.0081 (10)	0.3445 (4)	1.3637 (4)	0.079 (2)	
H8A	0.1469	0.3190	1.3798	0.118*	
H8B	−0.1046	0.3211	1.4069	0.118*	
H8C	0.0177	0.4029	1.3763	0.118*	
C9	0.4833 (8)	0.4345 (3)	0.8726 (4)	0.0420 (14)	
H9	0.5945	0.4587	0.9121	0.050*	
C10	0.7230 (8)	0.4779 (3)	0.6336 (4)	0.0528 (17)	
N1	0.4996 (7)	0.4345 (3)	0.7738 (3)	0.0486 (13)	
N2	0.6925 (7)	0.4696 (3)	0.7372 (3)	0.0530 (14)	
H2N	0.7890	0.4848	0.7866	0.064*	
N3	0.5579 (7)	0.4547 (3)	0.5733 (3)	0.0652 (15)	
H3NA	0.4296	0.4346	0.5911	0.078*	
H3NB	0.5698	0.4611	0.5043	0.078*	
O1	0.4483 (5)	0.4339 (2)	1.0882 (2)	0.0512 (12)	
H1O	0.4206	0.4284	1.1511	0.077*	
O2	0.1182 (6)	0.3677 (2)	1.1904 (3)	0.0550 (10)	
S1	0.9596 (2)	0.51784 (12)	0.58949 (9)	0.0666 (5)	
C11	0.2184 (8)	0.6114 (3)	0.8747 (4)	0.0383 (13)	
C12	0.3909 (9)	0.6490 (4)	0.8233 (4)	0.0451 (15)	
C13	0.5576 (9)	0.6839 (4)	0.8776 (5)	0.0503 (17)	
H13	0.6739	0.7091	0.8430	0.060*	
C14	0.5540 (9)	0.6818 (3)	0.9861 (5)	0.0528 (17)	
H14	0.6696	0.7051	1.0236	0.063*	
C15	0.3844 (9)	0.6461 (4)	1.0377 (4)	0.0427 (15)	
H15	0.3829	0.6460	1.1101	0.051*	
C16	0.2128 (8)	0.6097 (3)	0.9825 (4)	0.0353 (13)	
C17	0.5280 (10)	0.6936 (4)	0.6567 (4)	0.071 (2)	
H17A	0.6758	0.6724	0.6673	0.086*	
H17B	0.5247	0.7512	0.6779	0.086*	
C18	0.4616 (12)	0.6855 (4)	0.5436 (4)	0.102 (3)	
H18A	0.4782	0.6290	0.5219	0.152*	
H18B	0.5537	0.7203	0.5018	0.152*	
H18C	0.3106	0.7020	0.5353	0.152*	
C19	−0.2344 (8)	0.5427 (3)	1.2735 (4)	0.0509 (16)	
C20	0.0220 (8)	0.5732 (3)	1.0337 (4)	0.0389 (15)	
H20	−0.0841	0.5461	0.9940	0.047*	
N4	−0.0013 (6)	0.5781 (3)	1.1322 (3)	0.0391 (11)	
N5	−0.1924 (6)	0.5445 (3)	1.1704 (3)	0.0479 (13)	
H5N	−0.2845	0.5167	1.1315	0.058*	
N6	−0.0761 (8)	0.5723 (3)	1.3345 (3)	0.0693 (16)	
H6NA	0.0549	0.5759	1.3077	0.083*	
H6NB	−0.0989	0.5638	1.4014	0.083*	
O3	0.0473 (5)	0.5768 (2)	0.8190 (2)	0.0515 (11)	
H3O	0.0650	0.5791	0.7552	0.077*	
O4	0.3715 (6)	0.6459 (2)	0.7167 (3)	0.0589 (11)	
S2	−0.4755 (2)	0.50462 (12)	1.31643 (9)	0.0625 (5)	

### Atomic displacement parameters (Å^2^)

**Table:**

	*U*^11^	*U*^22^	*U*^33^	*U*^12^	*U*^13^	*U*^23^
C1	0.034 (3)	0.040 (4)	0.038 (3)	−0.002 (3)	0.004 (3)	0.000 (3)
C2	0.040 (3)	0.048 (4)	0.039 (3)	0.001 (3)	0.009 (3)	0.005 (3)
C3	0.036 (3)	0.050 (4)	0.059 (4)	−0.006 (3)	0.012 (3)	0.001 (3)
C4	0.046 (4)	0.059 (5)	0.064 (4)	−0.011 (3)	−0.003 (3)	0.001 (3)
C5	0.054 (4)	0.044 (4)	0.045 (3)	−0.003 (3)	−0.003 (3)	0.004 (3)
C6	0.037 (3)	0.041 (4)	0.037 (3)	0.002 (3)	−0.002 (2)	0.000 (3)
C7	0.061 (4)	0.057 (5)	0.068 (5)	−0.002 (3)	0.032 (3)	0.020 (4)
C8	0.090 (5)	0.096 (7)	0.051 (4)	−0.002 (4)	0.028 (4)	0.014 (4)
C9	0.038 (3)	0.049 (4)	0.040 (3)	−0.002 (3)	−0.001 (2)	0.000 (3)
C10	0.047 (3)	0.077 (5)	0.035 (3)	−0.010 (3)	0.002 (3)	−0.005 (3)
N1	0.048 (3)	0.069 (4)	0.029 (3)	−0.006 (3)	0.007 (2)	0.003 (3)
N2	0.048 (3)	0.084 (4)	0.027 (2)	−0.011 (3)	0.001 (2)	0.002 (2)
N3	0.062 (3)	0.105 (5)	0.029 (3)	−0.030 (3)	0.000 (2)	0.002 (3)
O1	0.049 (2)	0.077 (3)	0.027 (2)	−0.016 (2)	0.0058 (18)	−0.005 (2)
O2	0.060 (3)	0.068 (3)	0.037 (2)	−0.010 (2)	0.0164 (19)	0.004 (2)
S1	0.0488 (9)	0.1187 (16)	0.0325 (8)	−0.0175 (10)	0.0068 (7)	0.0012 (10)
C11	0.038 (3)	0.039 (4)	0.038 (3)	−0.001 (3)	0.004 (3)	−0.008 (3)
C12	0.043 (3)	0.053 (4)	0.039 (3)	−0.007 (3)	0.014 (3)	−0.001 (3)
C13	0.037 (4)	0.054 (4)	0.060 (4)	−0.005 (3)	0.015 (3)	0.009 (3)
C14	0.043 (3)	0.047 (4)	0.068 (4)	−0.008 (3)	0.007 (3)	0.001 (3)
C15	0.045 (4)	0.041 (4)	0.042 (3)	0.003 (3)	−0.007 (3)	−0.005 (3)
C16	0.034 (3)	0.034 (4)	0.037 (3)	0.002 (3)	0.003 (2)	0.001 (3)
C17	0.081 (4)	0.077 (5)	0.056 (4)	−0.013 (4)	0.038 (4)	0.009 (4)
C18	0.131 (6)	0.123 (7)	0.050 (4)	0.001 (5)	0.039 (4)	0.008 (4)
C19	0.048 (3)	0.072 (5)	0.032 (3)	0.000 (3)	0.002 (3)	0.003 (3)
C20	0.038 (3)	0.050 (4)	0.030 (3)	0.006 (3)	−0.002 (2)	−0.002 (3)
N4	0.032 (2)	0.051 (3)	0.034 (3)	−0.006 (2)	−0.0008 (19)	0.005 (2)
N5	0.041 (3)	0.072 (4)	0.031 (2)	−0.010 (2)	−0.0018 (19)	0.000 (2)
N6	0.063 (3)	0.113 (5)	0.032 (3)	−0.025 (3)	−0.003 (3)	−0.004 (3)
O3	0.051 (2)	0.070 (3)	0.034 (2)	−0.017 (2)	0.0040 (18)	−0.0036 (19)
O4	0.063 (3)	0.067 (3)	0.047 (2)	−0.013 (2)	0.019 (2)	0.001 (2)
S2	0.0463 (9)	0.1101 (15)	0.0313 (8)	−0.0101 (10)	0.0066 (6)	0.0014 (9)

### Geometric parameters (Å, °)

**Table:**

C1—O1	1.347 (5)	C11—O3	1.378 (5)
C1—C6	1.381 (6)	C11—C12	1.382 (6)
C1—C2	1.409 (6)	C11—C16	1.385 (6)
C2—O2	1.367 (6)	C12—C13	1.351 (7)
C2—C3	1.375 (7)	C12—O4	1.375 (6)
C3—C4	1.379 (7)	C13—C14	1.395 (8)
C3—H3	0.9300	C13—H13	0.9300
C4—C5	1.387 (6)	C14—C15	1.356 (6)
C4—H4	0.9300	C14—H14	0.9300
C5—C6	1.399 (6)	C15—C16	1.389 (7)
C5—H5	0.9300	C15—H15	0.9300
C6—C9	1.460 (6)	C16—C20	1.459 (6)
C7—O2	1.426 (5)	C17—O4	1.447 (5)
C7—C8	1.480 (8)	C17—C18	1.513 (8)
C7—H7A	0.9700	C17—H17A	0.9700
C7—H7B	0.9700	C17—H17B	0.9700
C8—H8A	0.9600	C18—H18A	0.9600
C8—H8B	0.9600	C18—H18B	0.9600
C8—H8C	0.9600	C18—H18C	0.9600
C9—N1	1.273 (6)	C19—N6	1.327 (6)
C9—H9	0.9300	C19—N5	1.350 (5)
C10—N3	1.319 (6)	C19—S2	1.684 (5)
C10—N2	1.351 (5)	C20—N4	1.277 (6)
C10—S1	1.676 (5)	C20—H20	0.9300
N1—N2	1.386 (5)	N4—N5	1.374 (5)
N2—H2N	0.8964	N5—H5N	0.8736
N3—H3NA	0.8753	N6—H6NA	0.8703
N3—H3NB	0.8958	N6—H6NB	0.8816
O1—H1O	0.8316	O3—H3O	0.8286
			
O1—C1—C6	119.7 (4)	O3—C11—C12	120.1 (5)
O1—C1—C2	120.0 (5)	O3—C11—C16	119.3 (4)
C6—C1—C2	120.3 (5)	C12—C11—C16	120.6 (5)
O2—C2—C3	126.7 (5)	C13—C12—O4	126.2 (5)
O2—C2—C1	113.5 (5)	C13—C12—C11	120.3 (5)
C3—C2—C1	119.8 (5)	O4—C12—C11	113.5 (5)
C2—C3—C4	120.6 (5)	C12—C13—C14	119.5 (5)
C2—C3—H3	119.7	C12—C13—H13	120.3
C4—C3—H3	119.7	C14—C13—H13	120.3
C3—C4—C5	119.6 (5)	C15—C14—C13	120.9 (5)
C3—C4—H4	120.2	C15—C14—H14	119.5
C5—C4—H4	120.2	C13—C14—H14	119.5
C4—C5—C6	121.0 (5)	C14—C15—C16	120.0 (5)
C4—C5—H5	119.5	C14—C15—H15	120.0
C6—C5—H5	119.5	C16—C15—H15	120.0
C1—C6—C5	118.7 (5)	C11—C16—C15	118.7 (5)
C1—C6—C9	120.0 (5)	C11—C16—C20	118.8 (5)
C5—C6—C9	121.3 (5)	C15—C16—C20	122.4 (5)
O2—C7—C8	108.4 (5)	O4—C17—C18	107.0 (5)
O2—C7—H7A	110.0	O4—C17—H17A	110.3
C8—C7—H7A	110.0	C18—C17—H17A	110.3
O2—C7—H7B	110.0	O4—C17—H17B	110.3
C8—C7—H7B	110.0	C18—C17—H17B	110.3
H7A—C7—H7B	108.4	H17A—C17—H17B	108.6
C7—C8—H8A	109.5	C17—C18—H18A	109.5
C7—C8—H8B	109.5	C17—C18—H18B	109.5
H8A—C8—H8B	109.5	H18A—C18—H18B	109.5
C7—C8—H8C	109.5	C17—C18—H18C	109.5
H8A—C8—H8C	109.5	H18A—C18—H18C	109.5
H8B—C8—H8C	109.5	H18B—C18—H18C	109.5
N1—C9—C6	121.9 (5)	N6—C19—N5	115.6 (4)
N1—C9—H9	119.0	N6—C19—S2	124.6 (4)
C6—C9—H9	119.0	N5—C19—S2	119.8 (4)
N3—C10—N2	116.4 (4)	N4—C20—C16	120.9 (5)
N3—C10—S1	124.1 (4)	N4—C20—H20	119.5
N2—C10—S1	119.5 (4)	C16—C20—H20	119.5
C9—N1—N2	114.1 (5)	C20—N4—N5	115.3 (4)
C10—N2—N1	119.6 (4)	C19—N5—N4	121.5 (4)
C10—N2—H2N	125.4	C19—N5—H5N	115.4
N1—N2—H2N	115.0	N4—N5—H5N	122.6
C10—N3—H3NA	128.8	C19—N6—H6NA	116.8
C10—N3—H3NB	119.0	C19—N6—H6NB	113.8
H3NA—N3—H3NB	112.1	H6NA—N6—H6NB	122.9
C1—O1—H1O	108.4	C11—O3—H3O	113.3
C2—O2—C7	119.6 (4)	C12—O4—C17	117.2 (4)
			
O1—C1—C2—O2	0.0 (7)	O3—C11—C12—C13	179.2 (5)
C6—C1—C2—O2	179.3 (5)	C16—C11—C12—C13	0.7 (8)
O1—C1—C2—C3	−179.1 (5)	O3—C11—C12—O4	−1.1 (7)
C6—C1—C2—C3	0.1 (8)	C16—C11—C12—O4	−179.6 (4)
O2—C2—C3—C4	−178.7 (5)	O4—C12—C13—C14	−179.8 (5)
C1—C2—C3—C4	0.3 (8)	C11—C12—C13—C14	−0.1 (8)
C2—C3—C4—C5	−0.9 (8)	C12—C13—C14—C15	−0.8 (8)
C3—C4—C5—C6	1.1 (8)	C13—C14—C15—C16	1.2 (8)
O1—C1—C6—C5	179.3 (4)	O3—C11—C16—C15	−178.9 (5)
C2—C1—C6—C5	0.1 (7)	C12—C11—C16—C15	−0.3 (7)
O1—C1—C6—C9	−0.1 (7)	O3—C11—C16—C20	−1.7 (7)
C2—C1—C6—C9	−179.4 (5)	C12—C11—C16—C20	176.8 (5)
C4—C5—C6—C1	−0.7 (8)	C14—C15—C16—C11	−0.6 (8)
C4—C5—C6—C9	178.7 (5)	C14—C15—C16—C20	−177.6 (5)
C1—C6—C9—N1	179.8 (5)	C11—C16—C20—N4	−172.5 (5)
C5—C6—C9—N1	0.4 (8)	C15—C16—C20—N4	4.5 (8)
C6—C9—N1—N2	−177.9 (4)	C16—C20—N4—N5	177.3 (4)
N3—C10—N2—N1	2.9 (8)	N6—C19—N5—N4	−2.6 (8)
S1—C10—N2—N1	−178.2 (4)	S2—C19—N5—N4	177.4 (4)
C9—N1—N2—C10	−175.9 (5)	C20—N4—N5—C19	177.6 (5)
C3—C2—O2—C7	2.3 (8)	C13—C12—O4—C17	−8.7 (8)
C1—C2—O2—C7	−176.8 (5)	C11—C12—O4—C17	171.6 (5)
C8—C7—O2—C2	179.8 (5)	C18—C17—O4—C12	−175.6 (5)

### Hydrogen-bond geometry (Å, °)

**Table:**

*D*—H···*A*	*D*—H	H···*A*	*D*···*A*	*D*—H···*A*
O1—H1O···S2^i^	0.83	2.53	3.180 (4)	135
O3—H3O···S1^ii^	0.83	2.43	3.143 (4)	145
N2—H2N···O3^i^	0.90	2.20	2.954 (6)	142
N5—H5N···O1^ii^	0.87	2.17	3.009 (5)	160
N3—H3NB···S2^iii^	0.90	2.53	3.403 (4)	166
N6—H6NB···S1^iv^	0.88	2.55	3.398 (5)	161

Symmetry codes: (i) *x*+1, *y*, *z*; (ii) *x*−1, *y*, *z*; (iii) *x*+1, *y*, *z*−1; (iv) *x*−1, *y*, *z*+1.
